# A topology optimization algorithm for magnetic structures based on a hybrid FEM–BEM method utilizing the adjoint approach

**DOI:** 10.1038/s41598-021-04246-z

**Published:** 2022-01-21

**Authors:** Gregor Wautischer, Claas Abert, Florian Bruckner, Florian Slanovc, Dieter Suess

**Affiliations:** 1 Faculty of Physics, University of Vienna, Vienna, Austria; 2 Research Platform MMM Mathematics-Magnetism-Materials, University of Vienna, Vienna, Austria

**Keywords:** Applied physics, Condensed-matter physics

## Abstract

A method to optimize the topology of hard as well as soft magnetic structures is implemented using the density approach for topology optimization. The stray field computation is performed by a hybrid finite element–boundary element method. Utilizing the adjoint approach the gradients necessary to perform the optimization can be calculated very efficiently. We derive the gradients using a “first optimize then discretize” scheme. Within this scheme, the stray field operator is self-adjoint allowing to solve the adjoint equation by the same means as the stray field calculation. The capabilities of the method are showcased by optimizing the topology of hard as well as soft magnetic thin film structures and the results are verified by comparison with an analytical solution.

## Introduction

In many applications like magnetic sensors, magnetic storage, electric machines and so on, magnetic fields with specific spatial profiles are exploited in order to achieve a specific goal. The properties of magnetic fields are defined by the properties of the involved magnetic structures. Most commonly, in order to obtain a certain magnetic field, the geometry of a magnetic structure is optimized. This can e.g. be the geometry of a permanent magnet producing a well defined magnetic field, or a soft magnetic structure shaping the field possibly generated by an electric current. The process of optimizing the geometry of these structures can be very time consuming. A common approach is called geometry parameterization where the structure’s geometry is parameterized with relatively few parameters and the parameter region is investigated until an optimal solution is found. Geometry parameterization is one representative of a group of techniques summarized as shape optimization^1^. Another approach is to directly optimize the distribution of material within a given domain. This approach is called topology optimization^2^. It drastically increases the degrees of freedom (dofs) but allows for possibly better, geometric realizations of the considered system, independent of any introduced parameterizations. Successful applications of topology optimization of magnetic structures include the optimization of magnetic recording write heads^3^, of rotors of brushless DC motors^4^ and of electromagnetic actuators^5,6^.

In this paper a method to optimize the topology of magnetic structures is presented. The presented method is based on a highly efficient hybrid finite element–boundary element method (FEM–BEM) approach^7^ solving the magnetostatic Maxwell’s problem. In contrast to already presented finite element^3,8^ and finite differences^9^ algorithms using a FEM–BEM approach has the advantage that only the regions of interest need to be discretized reducing the dofs dramatically. Furthermore, in order to efficiently calculate gradients during optimization, the adjoint approach is utilized. Our method takes the B/H curve of a hard or soft magnetic material linearized at the working point as input and is able to include external fields generated by electric coils. After optimization, the result is an optimized magnetic structure and its stray field dependent magnetization state.

The algorithm is implemented on the basis of magnum.fe^10^ which in turn utilizes the finite element library FEniCS^11^. In particular FEniCS is used for the definition of the objective functionals and to automatically differentiate the involved partial derivatives.

The paper is structured as follows. In the first section the solution of the forward problem–the calculation of the stray field of a magnetic structure—is presented. In the following section the density approach to topology optimization is introduced. After that the gradient necessary to perform the optimization and the adjoint equation are introduced. Thereafter details of the numerical optimization are discussed and numerical experiments showcasing the capabilities of the method are presented. A detailed derivation of the gradient using the adjoint approach within a “first optimize then discretize” scheme is given in the appendix.

## Forward problem

The stray field of a magnetic body consisting of a magnetic material with linear magnetic susceptibility $\chi$ and possibly a remanence magnetization ${\varvec{M}}_\text {r}$ within an external magnetic field ${\varvec{H}}_{\text {ext}}$, can be calculated starting from the magnetostatic Maxwell’s equation. Using the reduced scalar potential formulation the total magnetic field ${\varvec{H}}$ is split into an external part ${\varvec{H}}_{\text {ext}}$ (created via currents outside of $\Omega _{\text {m}}$) and the curl-free induced magnetic field ${\varvec{H}}_{\text {d}}$. Expressing the induced field as gradient of a scalar potential *u*, ${\varvec{H}}_{\text {d}} = -\varvec{\nabla }u$ finally yields

1 $$\begin{aligned} \varvec{\nabla }\cdot \left[ \left( 1+\chi \right) \left( \varvec{\nabla }u - {\varvec{H}}_{\text {ext}}\right) - {\varvec{M}}_\text {r}\right] = 0. \end{aligned}$$

In order to use the hybrid FEM–BEM approach, next ${\mathbb {R}}^3$ is divided into a region $\Omega _{\text {m}}$ containing all magnetic material and a region ${\mathbb {R}}^3 {\backslash }\Omega _\text {m}$. Outside $\Omega _m$ Eq. (1) then reduces to

2 $$\begin{aligned} \Delta u = 0 \quad \text {in} \quad {\mathbb {R}}^3 {\backslash }\Omega _m \end{aligned}$$

and the following boundary and jump conditions apply

3 $$\begin{aligned} \left[ u\right] _{\partial \Omega _{\text {m}}}&= \,0 \end{aligned}$$

4 $$\begin{aligned} \left[ \left( 1+\chi \right) \frac{\partial u}{\partial {\varvec{n}}}\right] _{\partial \Omega _{\text {m}}}&= \,\left[ \chi \right] _{\partial \Omega _{\text {m}}}{\varvec{n}}\cdot {\varvec{H}}_{\text {ext}}+ {\varvec{n}}\cdot {\varvec{M}}_{\text {r}} \end{aligned}$$

5 $$\begin{aligned}&u\left( {\varvec{r}}\right) \rightarrow {\mathcal {O}}\left( \frac{1}{\left| {\varvec{r}}\right| }\right) \quad \text {for} \quad \left| {\varvec{r}}\right| \rightarrow \infty \text {,} \end{aligned}$$

where ${\varvec{n}}$ is the outward pointing normal vector and $\left[ \cdot \right] _{\partial \Omega }$ denotes the jump over the boundary $\partial \Omega$. The resulting system is solved using a hybrid FEM–BEM algorithm^7^.

## Topology optimization

Topology optimization tries to find, within a predefined spatial domain $\Omega _{\text {opt}}$, the optimal material distribution with respect to a design goal. This can be achieved using the density approach for topology optimization^12^. Here, a scalar indicator function

6 $$\begin{aligned} \rho \left( {\varvec{x}}\right) \in \left[ 0,1\right] \text {,}\quad \text {where}\quad \rho \left( {\varvec{x}}\right) = {\left\{ \begin{array}{ll} 0\text {: no material}\\ 1\text {: material} \end{array}\right. } \end{aligned}$$

is introduced, transforming the remanence magnetization ${\varvec{M}}_{\text {r}}\rightarrow {\varvec{M}}_{\text {r}}\left( \rho \right) = \rho ^p {\varvec{M}}_{\text {r}}$ as well as the susceptibility $\chi \rightarrow \chi \left( \rho \right) = \rho ^p\chi$. Here, *p* is a parameter introduced in order to penalize intermediate values of $0 \le \rho \le 1$ that are allowed during optimization^12^. However, it turns out that the impact of *p* is more complex as is investigated for each presented numerical experiment (see “Numerical experiments” section).

The forward problem then reads

7 $$\begin{aligned} F\left( u, \rho \right) =\varvec{\nabla }\cdot \left[ \left( 1+\rho ^p\chi \right) \left( \varvec{\nabla }u -{\varvec{H}}_{\text {ext}}\right) - \rho ^p{\varvec{M}}_\text {r}\right] = 0, \end{aligned}$$

where *u* is an at least twice and $\rho$ an at least once differentiable function. Note, that after transformation into its weak form and shifting of the divergence operator onto the test function by application of partial integration, these conditions are relaxed and after discretization piecewise linear basis function $\left( {\mathcal {P}}_1\right)$ can be used for the scalar potential *u* and the scalar indicator function $\rho$ only has to be constant within each element.

The numerical optimization is performed by casting the design goal into an objective functional ${\hat{J}}\left( \rho \right) = J\left( {\varvec{H}}\left( \rho \right) , \rho \right)$ and minimizing it using an iterative, gradient based optimization algorithm.

## Adjoint approach

In order to efficiently minimize the reduced objective functional ${\hat{J}}\left( \rho \right)$ containing the design goal, its gradient with respect to the design variable $\rho$, $\varvec{\nabla }_{\rho }{\hat{J}}$ is needed. This is where the adjoint approach comes into play, offering a very efficient method to calculate the gradient. As derived in detail in the appendix (“Appendix” section) within a “first optimize then discretize” scheme, the gradient is given by

8 $$\begin{aligned} \varvec{\nabla }_{\rho }{\hat{J}} = \left( p\rho ^{p-1}\left[ \chi \left( \varvec{\nabla }u-{\varvec{H}}_{\text {ext}}\right) - {\varvec{M}}_{\text {r}}\right] \right) \cdot \varvec{\nabla }\lambda + {\mathcal {R}}\left( \frac{\partial J}{\partial \rho }\right) \text {,} \end{aligned}$$

where $\lambda$ is the adjoint variable that is obtained by solving the adjoint equation

9 $$\begin{aligned} \varvec{\nabla }\cdot \left( \left( 1+\rho ^p\chi \right) \varvec{\nabla }\lambda -{\mathcal {R}}\left( \frac{\partial J}{\partial {\varvec{H}}}\right) \right) = 0 \end{aligned}$$

with boundary condition

10 $$\begin{aligned} \lambda \left( {\varvec{r}}\right) \rightarrow {\mathcal {O}}\left( \frac{1}{\left| {\varvec{r}}\right| }\right) \quad \text {for} \quad \left| {\varvec{r}}\right| \rightarrow \infty \text {.} \end{aligned}$$

Here ${\mathcal {R}}$ are the Riesz representers of the corresponding functionals as introduced in “Appendix” section. Note, that the adjoint equation has the same form as the forward problem. Therefore, the same algorithm can be utilized to solve the forward as well as the adjoint equation.

## Numerical experiments

**Figure 1:**
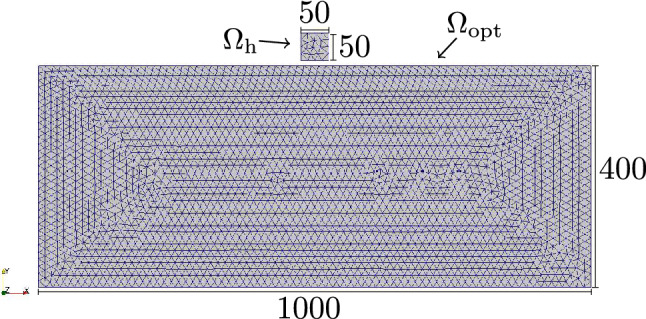
Mesh and geometry used for the numerical experiments. Dimensions are given in µm. The thickness (*z*-dimension) of the thin film is 12.5 µm. The field box $\Omega _\text {h}$ consists of 91 cells and the optimization region $\Omega _{\text {opt}}$ consists of 16505 cells.

In the following, the topology of thin film magnetic flux guide concentrators is optimized towards maximization of the vertical (*y*-)component of the magnetic field $H_y$ inside the target domain $\Omega _{\text {h}}$. The objective functional that is to be minimized reads ${\hat{J}} = -\int _{\Omega _{\text {h}}} H_y\,\text {d}V$. The geometry and the mesh used is depicted in Fig. 1 where also the the optimization domain ($\Omega _{\text {opt}}$) and the domain in which the magnetic field is evaluated ($\Omega _{\text {h}}$) is specified. Note that $\Omega _{\text {m}} = \Omega _{\text {opt}} \cup \Omega _{\text {h}}$. Note, that we use an irregular mesh in order to avoid any solution bias introduced by a given regularity. The presented numerical experiments include the usage of hard as well as soft magnetic material.

The potential *u* and the adjoint variable $\lambda$ are calculated using piecewise linear basis functions $\left( {\mathcal {P}}_1\right)$ and the scalar indicator function $\rho$ as well as $\chi$, ${\varvec{M}}_{\text {r}}$, ${\varvec{H}}_{\text {ext}}$ and the derived strayfield $-\varvec{\nabla }u$ and $\varvec{\nabla }\lambda$ are constant within each element.

The optimizations are performed using the Scipy implementation of the limited-memory Broyden-Fletcher-Goldfarb-Shanno minimization algorithm with bounds (L-BFGS-B)^13^. Note, that as shown in^14^, common ready-to-use optimization algorithms such as this, are constructed to only receive coefficient vectors as input and internally use the euclidean ($\ell ^2$) inner product to perform all necessary operations (e.g. approximation of the Hessian). Since here an optimization in a Hilbert spaces with the $L^2$ inner product is performed, this leads to mesh dependent convergence rates. To prevent this, there exists the python package Moola^15^ that internally uses the correct inner product during optimization. However, in order to perform topology optimization bounds on the optimization variable $\rho$ have to be enforced since $0 \le \rho \le 1$ and so far, there is no optimization algorithm offered by Moola enabling bounds on the optimization variable. Furthermore, note that the $L^2$ Riesz representer of the gradient is independent of the flux concentrator’s geometrical dimensions. However, the objective functional is not since it includes and integration over the volume $\Omega _{\text {h}}$. For the chosen geometry dimensions the gradient size is relatively large compared to the size of the objective functional. This leads to the L-BFGS-B algorithm terminating prematurely with problems during the line search, simply returning the initial distribution of $\rho$. This is since the Armijo rule, one of the Wolfe conditions necessary to end the line search is never met. The objective functional value exposed to the optimization algorithm, is therefore scaled to avoid this problem. This scaling does not alter the results since internally, the L-BFGS-B algorithm performs a quasi-Newton method on the gradient and the function value is only used during the line search.

For results of optimizations that terminate with dofs of $\rho$ at intermediate values $0\le \rho \le 1$ a brute force regularization is applied where all dofs with $\rho < 0.5$ are set to 0 and all dofs with $\rho \ge 0.5$ are set to 1.

### Permanent magnet optimization

**Figure 2:**
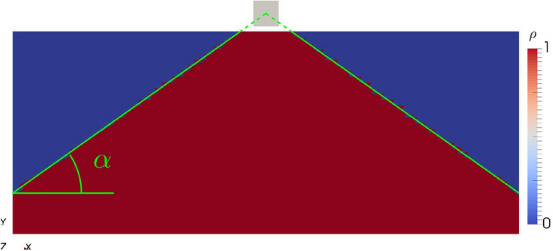
Optimized topology for the ideal permanent magnet and comparison with the analytical solution (green line).

**Figure 3:**
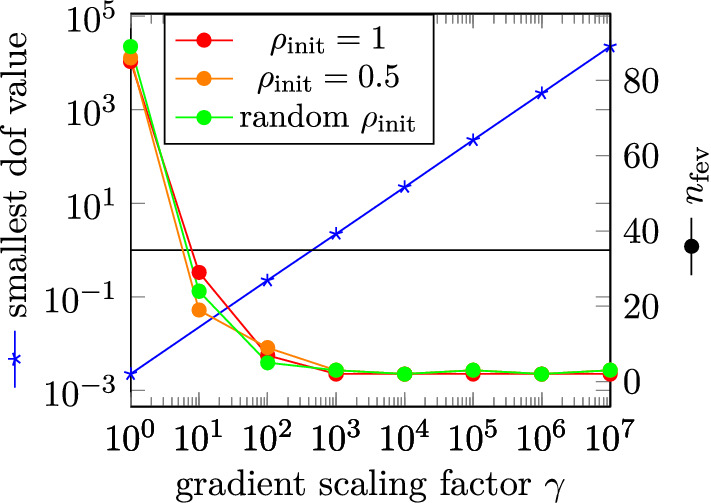
Dependence of $n_{\text {fev}}$ on $\gamma$ and size of the gradient’s smallest dof value. The black horizontal line represents a value of 1.

First, the topology for an ideal permanent magnet with susceptibility $\chi = 0$, remanence magnetization $M_{\text {r}} = 280000$ A/m and the anisotropy axis chosen as the *y*-axis is optimized towards maximization of the vertical (*y*-)component of the magnetic field $H_y$ inside the domain $\Omega _h$, as introduced above.

As presented in^8^ for a point like target domain, the optimal topology can be derived analytically by considering the magnetization inside $\Omega _{\text {opt}}$ as being comprised of single dipoles $\varvec{\mu }$. A dipole at a vertical (*y*-)distance produces a positive flux $B_y\left( {\varvec{x}}\right)$ at a point ${\varvec{x}}$ for an horizontal (*x*-)distance of

11 $$\begin{aligned} x < \sqrt{2}y\text {.} \end{aligned}$$

The surface condition $x=\sqrt{2}y$ therefore shows that the analytical solution is a cone with an opening angle of $\alpha \approx 35.26^{\circ }$. Note that the assumption of a point like target domain is still valid, since the changes due to the finite size of $\Omega _{\text {h}}$ are too small to be resolved by the chosen mesh. The analytical solution is shown in Fig. 2, where it is compared to the optimal numerical solution.

For the ideal permanent magnet, the optimized topology, as shown in Fig. 2, produces a mean field ${\bar{H}}_y = \int _{\Omega _{\text {h}}} H_y\,\text {d}V / V_{\Omega _{\text {h}}}$ of 22.83 mT. This solution is found independently of the initial distribution of the density functional $\rho _{\text {init}}$ for a penalty parameter of $p\le 4$. Furthermore, scaling the gradient has no influence on the obtained solution, but increasing the gradient size reduces the number of function evaluations $n_{\text {fev}}$ needed. This is since the size of the gradient determines how fast the dofs of $\rho$ are set to 0 or 1. This shows, that the optimization problem in the case of zero susceptibility is linear in $\rho$ as expected since for $\chi = 0$ the only source term in the forward problem (7) is given by $\rho ^p{\varvec{M}}_{\text {r}}$ where ${\varvec{M}}_{\text {r}}$ is constant. In Fig. 3 the value of the smallest dof in the gradient and $n_{\text {fev}}$ are plotted against the gradient scaling factor $\gamma$ for $p=1$. As soon as the smallest dof is larger 1 for $\gamma = 10^3$, all elements are set to 0 or 1 during the optimization, leaving no dofs with intermediate values of $\rho$ and $n_{\text {fev}}$ reaches a minimum of 2.

### Realistic hard magnetic material $\chi = 0.2$

**Figure 4:**
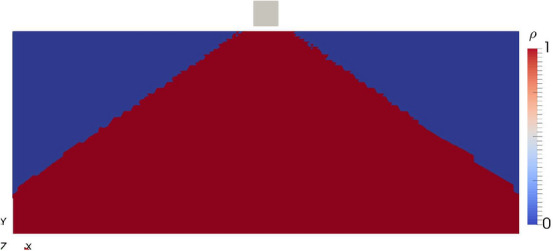
Optimized topology for the optimization with $\chi = 0.2$.

**Figure 5:**
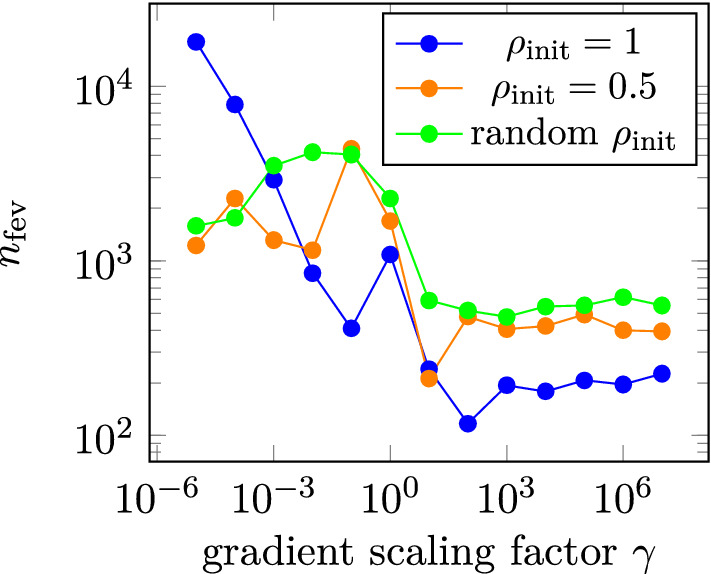
Dependence of the number of necessary function evaluations $n_{\text {fev}}$ on $\gamma$ with $p = 1$ for the optimization with $\chi = 0.2$.

Using a realistic hard magnetic material with a susceptibility of $\chi = 0.2$, the optimal solution found has a mean field of ${\bar{H}}_y$ of 22.35 mT. This smaller mean field value compared to the ideal permanent magnet is due the effect of demagnetization. The optimal topology found (Fig. 4) differs only slightly from the topology obtained from the ideal permanent magnet optimization.

The optimal solution is found independently of the initial distribution of the density functional $\rho$ for $p=1$. For larger *p*-values the solution gets worse showing, that the optimization is no longer independent of the parameter *p*. However, for $p\le 4$ the difference in the mean field is below 0.5%. Scaling the gradient again reduces the number of necessary function evaluations to perform the optimization $n_{\text {fev}}$. However, since the optimization problem is no longer linear in $\rho$, the number of function evaluations $n_{\text {fev}}$ needed is larger than for the ideal permanent magnet case. In Fig. 5$n_{\text {fev}}$ in dependence of the gradient scaling factor $\gamma$ for $p=1$ is shown. The optimization is fastest for a constant initial distribution of $\rho _{\text {init}} = 1$ where for $\gamma \ge 10$ $n_{\text {fev}} < 250$. Also, the optimization process terminates before all dofs of $\rho$ are set to 0 or 1, although the number of dofs with an intermediate value of $\rho$ is negligible for all simulations with $p=1$. It has been verified that no better solution is found by scaling the gradient down to $\gamma = 10^{-5}$. Note, that the topology obtained from the optimization using an ideal permanent magnetic material, for a realistic hard magnetic material with $\chi = 0.2$ produces a magnetic field that is only negligibly smaller (<0.1‰) than the optimal topology obtained using the realistic hard magnetic material during optimization. This confirms, that $\chi = 0$ is a good assumption for a permanent magnetic structure.

### Optimization of a soft magnetic structure

**Table 1:** Mean field values $\bar{H}_y$ and increase with respect to the full plate for the optimized topologies.

$\chi$	$\bar{H}_y$ (mT)	Increase (%)
$10^2$	14.65	52.25
$10^3$	21.95	84.38
$10^4$	23.34	91.23

Next, the topology of a soft magnetic thin film is optimized to act as a magnetic flux guide concentrator maximizing the magnetic flux in vertical (*y*-)direction inside $\Omega _{\text {h}}$. Such a structure can be used to e.g. improve the magnetic field detection limits of spin valve sensors^16^ by ehancing the external field at the sensor position.

The optimization is performed for three

**Figure 6:**
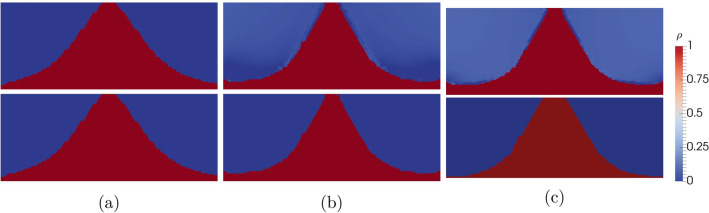
Optimized topologies before (top row) and after regularization (bottom row) for (**a**) $\chi = 10^2$ (for $p = 5$) (**b**) $\chi = 10^3$ (for $p = 14$) and (**c**) $\chi = 10^4$ (for $p = 17$) after regularization for $\gamma = 10^{-4}$.

different values of $\chi$ of $10^2$, $10^3$ and $10^4$ with an external field of 10 mT applied. The optimized topologies for the three different $\chi$-values are shown in Fig. 6. The found structures show the same “tanga” shape as reported in^16^ as optimal design. While the optimal topology differs only slightly for $\chi = 10^3$ and $\chi = 10^4$, for $\chi = 10^2$ the topology is significantly broader. In fact, switching the optimal topologies of $\chi = 10^3$ and $\chi = 10^4$ changes the generated mean field by under $0.2\%$. The generated mean field and the increase with respect to the full plate ($\rho =1$ in $\Omega _{\text {opt}}$) is shown in Table 1.

**Figure 7:**
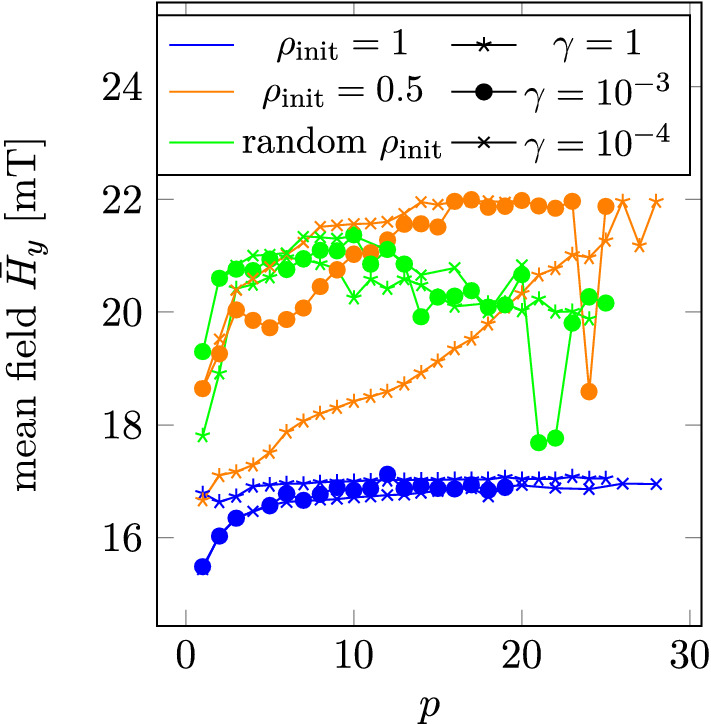
*p*-dependency for different initial distributions and gradient scaling factors $\gamma$ for the optimization performed for $\chi = 10^3$.

As opposed to the optimization of hard magnetic materials, the optimization using a soft magnetic material depends much more on the initial distribution of the density functional $\rho _{\text {init}}$ as well as on the *p*-parameter. In Fig. 7 the *p*-dependence of the mean field ${\bar{H}}_y$ for the regularized solutions for $\chi =10^3$ is plotted for different initial distributions of $\rho _{\text {init}}$ and for different values of the gradient scaling factor $\gamma$.

**Figure 8:**
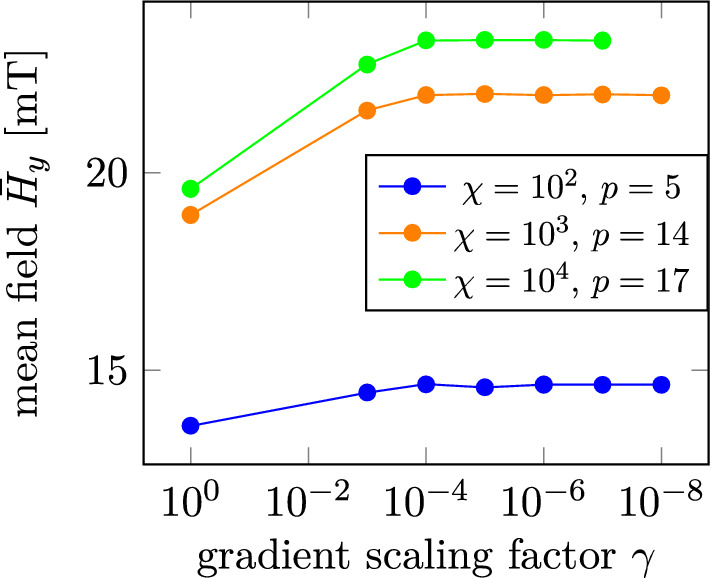
Dependency of the mean field ${\bar{H}}_y$ on $\gamma$ individual *p*-values.

The solution found for a constant $\rho _{\text {init}} = 1$ and a randomly initialized $\rho _{\text {init}}$ are clearly inferior to the solutions found with a constant $\rho _{\text {init}} = 0.5$. For $\rho _{\text {init}} = 0.5$ the mean field increases with increasing *p* until it plateaus. While decreasing $\gamma$ does not lead to a higher mean field, the *p*-value at which the mean field plateaus decreases with decreasing $\gamma$. At $\gamma = 1$ the plateau starts at $p = 26$ while for $\gamma = 10^{-4}$ the plateau value is 14. Further decreasing $\gamma$ does not alter the plateau value anymore. In fact, as shown in Fig. 8 also for single *p*-values, the mean field increases with decreasing $\gamma$ until a maximum is found for $10^{-4}$. Note, that for higher *p*-values than the plateau value, the mean field differs well under 1% from the mean field obtained by the optimization performed with the plateau value, except for individual outliers with very small mean field values for large *p*-values.

**Figure 9:**
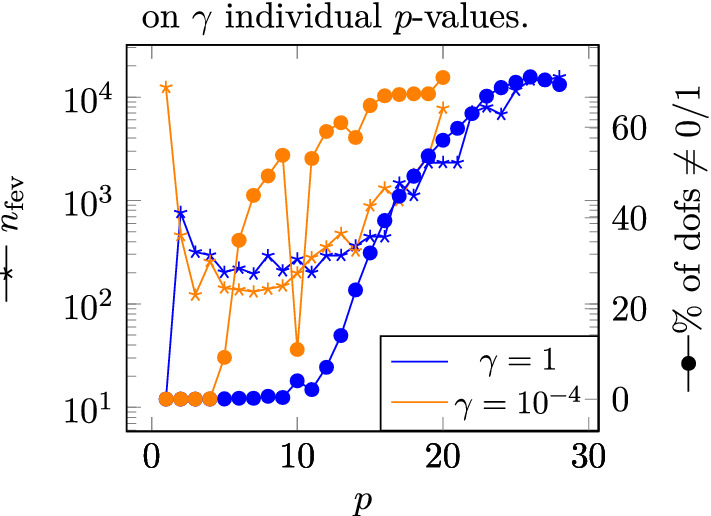
*p*-dependency of $n_{\text {fev}}$ and the percentage of dofs with intermediate values of $\rho$ after optimization for values of $\gamma$ for the optimization performed for $\chi = 10^3$.

**Figure 10:**
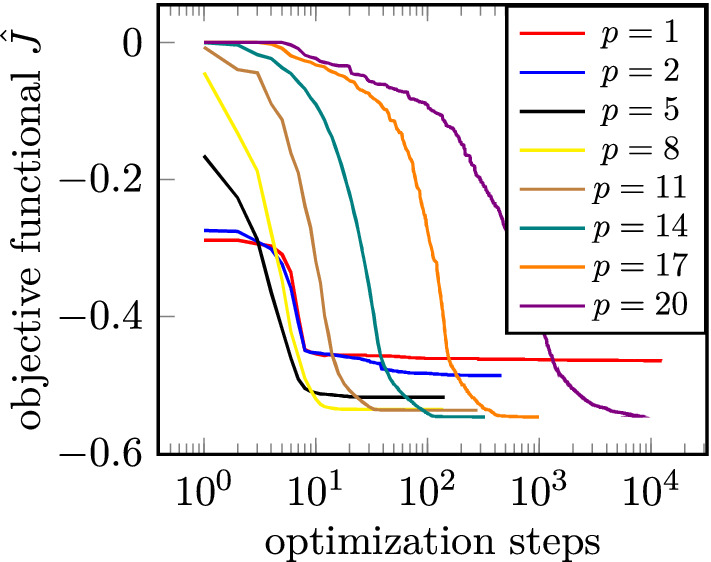
Iteration histories for selected values of *p* for a gradient scaling factor of $10^{-4}$.

In Fig. 9, the amount of dofs with intermediate values of $\rho$ after optimization is plotted in dependence of *p*. While the solutions with $p \le 4$ show no dofs with intermediate values of $\rho$, with increasing *p*, their number increases and reaches over 60% for the optimal solutions. This is in contrast to the original purpose of the penalization factor *p*. However, since the changes of the mean field ${\bar{H}}_y$ due to regularization is under 1% for all optimizations, the effect of the regularization is still negligible. This is since the remaining intermediate values are very close to 0 and 1 respectively. In Fig. 6 the optimized topologies are shown before and after regularization for comparison.

The dependence of the number of function evaluations necessary to perform the optimizations $n_{\text {fev}}$ on *p* is plotted in Fig. 9 for two different values of the gradient scaling factor. It can be seen $n_{\text {fev}}$ is lower with a smaller gradient scaling factor. With a gradient scaling factor of $10^{-4}$, the optimization with $p=14$ needs 325 $n_{\text {fev}}$ while with a gradient scaling factor of 1 for the optimization with $p=26$ $n_{\text {fev}} = 14631$. In Fig. 10 the iteration histories are plotted for selected values of *p* for a gradient scaling factor of $10^{-4}$. Note that the different starting values of the optimizations are due to factorization of $\rho$ by *p*.

For $\chi = 10^4$ the *p*-dependence of the mean field plateaus in the same way as for $\chi = 10^3$, although with a different plateau value of $p=17$ for a gradient scaling factor of $10^{-4}$. The dependency on the gradient scaling factor is also similar as shown in Fig. 8. For $\chi = 100$ the situation is slightly different. Here, for gradient scaling factors smaller $10^{-4}$ the mean field increases with increasing *p*-value until a maximum is found at $p=5$ and the mean field decreases when further increasing *p*.

## Conclusion

An algorithm to optimize the topology of magnetic structures based on a hybrid FEM–BEM method and the density approach for topology optimization has been presented. The gradients necessary to efficiently solve the optimization are obtained very efficiently using the adjoint approach. It has been shown that within a “first optimize then discretize” scheme the continuous forward operator is self-adjoint and therefore, the same method can be used to solve the forward as well as the adjoint problem.

The dependence on the *p*-parameter, originally introduced to penalize intermediate values of the density indicator function $0\le \rho \le 1$ has been investigated for the presented numerical experiments. For ideal permanent magnetic structures ($\chi = 0$) the linearity of the optimization problem can be utilized by enlarging the gradient. Scaling the gradient by a factor $>10^{3}$, with $p=1$ the optimization needs only two function evaluations and the found solution is in accordance with analytical expectations and shows no intermediate values of the indicator function $\rho$. For a realistic permanent magnetic material with $\chi = 0.2$ the optimized topology produces a magnetic field that differs by $<0.1$‰ from the topology found for the ideal permanent magnetic material. This shows that for thin films with negligible demagnetization, $\chi = 0$ is good approximation for a realistic permanent magnetic material with $\chi = 0.2$. Furthermore, the number of function evaluation needed using a realistic hard magnetic material is in the hundreds and hence much larger than the number of evaluations needed for the assumption of an ideal permanent magnetic material.

For the optimization of soft magnetic materials the optimal solutions are found for a gradient scaling factor $\le 10^{-4}$. The solutions found get better with increasing value of the *p*-parameter. For $\chi =10^2$ the best solution is found for $p=5$ and gets worse for larger *p*-values. For $\chi = 10^3$ and $\chi =10^4$ the solutions improve with *p* until it plateaus for $p\ge 14$ and $p\ge 17$ respectively. Note, that in contrast to its original purpose, the *p*-parameter does not decrease the number of dofs with intermediate values of $\rho$. On the contrary, the percentage of dofs with intermediate values of $\rho$ increases with increasing *p*. However, using a brute force regularization forcing all dofs with $\rho < 0.5$ to 0 and all dofs with $\rho \ge 0.5$ to 1 only negligibly impacts the field value created by the structures. The optimized topologies have a shape similar to the optimal shape found experimentally in^16^.

## Appendix

### Deriving the gradient $\varvec{\nabla }_{\rho }{\hat{J}}$ using the adjoint approach

In the following the adjoint approach is applied to obtain the gradient $\varvec{\nabla }_{\rho }{\hat{J}}$ of the reduced objective functional ${\hat{J}}\left( \rho \right)$ with respect to the design variable $\rho$. A “first optimize then discretize” scheme^17^ is used, within which the gradient is derived using continuous operators. These operators are, as will turn out, self-adjoint where the discrete forward operator of the hybrid FEM–BEM algorithm is not. This simplifies the numerics involved in solving the adjoint equation.

Following^17^, in order to derive the gradient $\varvec{\nabla }_{\rho }{\hat{J}}$ we consider the derivative

12 $$\begin{aligned} \frac{\text {d}{\hat{J}}}{\text {d}\rho } = \frac{\partial J}{\partial u}\frac{\text {d}u}{\text {d}\rho } + \frac{\partial J}{\partial \rho }\text {.} \end{aligned}$$

Here, $\frac{du}{d\rho }$ is the numerically challenging term since *u* depends on $\rho$ via the forward problem (7). In the discretized form, naive evaluation of $\frac{du}{d\rho }$ by means of finite difference would require $n_{\rho }$ solutions of the forward problem, where $n_{\rho }$ is the number of dofs of $\rho$. Looking at the derivative of the forward problem

13 $$\begin{aligned} \frac{\text {d}F}{\text {d}\rho } = \frac{\partial F}{\partial u}\frac{\text {d}u}{\text {d}\rho }+\frac{\partial F}{\partial \rho } = 0 \end{aligned}$$

the critical term can be expressed as

14 $$\begin{aligned} \frac{\text {d}u}{\text {d}\rho } = -\left( \frac{\partial F}{\partial u}\right) ^{-1}\frac{\partial F}{\partial \rho }\text {.} \end{aligned}$$

Inserting this into Eq. (12) gives

15 $$\begin{aligned} \frac{\text {d}{\hat{J}}}{\text {d}\rho } = -\frac{\partial J}{\partial u}\left( \frac{\partial F}{\partial u}\right) ^{-1}\frac{\partial F}{\partial \rho } + \frac{\partial J}{\partial \rho }\text {,} \end{aligned}$$

an expression for the derivative where $\frac{\text {d}u}{\text {d}\rho }$ does not appear explicitly anymore.

Consider now that the goal is to perform an optimization on $\rho$, an at least once differentiable element of a suitable Hilbert space *R*. This means, that an initial guess $\rho _0$ is updated iteratively using a suitable update candidate to obtain a new $\rho _i$ that further reduces the objective functional. Since the derivative $\frac{\text {d}{\hat{J}}}{\text {d}\rho }: R \rightarrow {\mathbb {R}}$ is a functional and therefore an element of the dual space $R^*$ of *R*, it is not a suitable candidate to be used as update during optimization. What is needed is an element of the primal space similar to $\rho$, the gradient $\varvec{\nabla }_{\rho }{\hat{J}} \in R$^14^.

Since within a finite element setting all function spaces are Hilbert spaces, the Riesz representation theorem can be used to obtain the gradient. The Riesz representation theorem states, that the dual space $H^*$ of a Hilbert space *H* is isometric to itself. This means that the identification $H = H^*$ can be done and for a functional $I \in H^*$ applied to $u \in H$ a unique element *w* of the primal space *H* can be found such that

16 $$\begin{aligned} I\left( u\right) {:}{=}\left( w, u\right) _H\quad \forall u \in H\text {,} \end{aligned}$$

where $\left( \cdot , \cdot \right) _H$ is the inner product defined on *H* and $w \in H$ is the Riesz representer of *I* with respect to the inner product on *H*, also denoted as $w = {\mathcal {R}}_H\left( I\right)$. Note that, for the presented example the appropriate inner product is the $L^2$ inner product that arises naturally when the forward problem is brought into its weak form by multiplication with a suitable test function. The specific reference is therefore dropped from this point on an each inner product as well as the Riesz representers are assumed to be with respect to $L^2$ if not stated otherwise.

The gradient $\varvec{\nabla }_{\rho }{\hat{J}} \in R$ can now be identified as the Riesz representer of the derivative $\frac{d{\hat{J}}}{d\rho }$^17^, pg. 99]

17 $$\begin{aligned} \frac{\text {d}{\hat{J}}}{\text {d}\rho }\left( s\right) = \left( \varvec{\nabla }_{\rho }{\hat{J}}, s\right) = -\left( {\mathcal {R}}\left( \frac{\partial J}{\partial u}\right) , \left( \frac{\partial F}{\partial u}\right) ^{-1}\frac{\partial F}{\partial \rho }s\right) + \Bigg ({\mathcal {R}}\left( \frac{\partial J}{\partial \rho }\right) , s\Bigg )\text {,} \end{aligned}$$

where also the Riesz representers of $\frac{\partial J}{\partial u}$ and $\frac{\partial J}{\partial \rho }$ have to be used. Using, that for an operator $A: H_1 \rightarrow H_2$ where $H_1$ and $H_2$ are Hilbert spaces, dual operator $A^*: H_2 \rightarrow H_1$ is defined as

18 $$\begin{aligned} \left( A^*\left( v\right) , u\right) = \left( v, A\left( u\right) \right) \quad \forall u \in H_1, v \in H_2, \end{aligned}$$

one can continue with

19 $$\begin{aligned} \left( \varvec{\nabla }_{\rho }{\hat{J}}, s\right) = -\left( \left( \frac{\partial F}{\partial \rho }\right) ^*\left( \frac{\partial F}{\partial u}\right) ^{-*}{\mathcal {R}}\left( \frac{\partial J}{\partial u}\right) , s\right) + \Bigg ({\mathcal {R}}\left( \frac{\partial J}{\partial \rho }\right) , s\Bigg ) \end{aligned}$$

and the gradient can be written as

20 $$\begin{aligned} \varvec{\nabla }_{\rho }{\hat{J}} = \left( \frac{\partial F}{\partial \rho }\right) ^*\left( \frac{\partial F}{\partial u}\right) ^{-*}{\mathcal {R}}\left( \frac{\partial J}{\partial u}\right) +{\mathcal {R}}\left( \frac{\partial J}{\partial \rho }\right) \text {.} \end{aligned}$$

The adjoint equation is now obtained by expressing the term $\left( \frac{\partial F}{\partial u}\right) ^{-*}{\mathcal {R}}\left( \frac{\partial J}{\partial u}\right)$ as a new variable $\lambda$ and rearranging to get

21 $$\begin{aligned} \left( \frac{\partial F}{\partial u}\right) ^{*}\lambda = {\mathcal {R}}\left( \frac{\partial J}{\partial u}\right) \text {.} \end{aligned}$$

This is the adjoint equation that can be used to calculate the adjoint variable $\lambda$ and obtain the gradient via

22 $$\begin{aligned} \varvec{\nabla }_{\rho }{\hat{J}} = \left( \frac{\partial F}{\partial \rho }\right) ^*\lambda +{\mathcal {R}}\left( \frac{\partial J}{\partial \rho }\right) \text {.} \end{aligned}$$

### Obtaining the dual operators

In order to calculate the gradient $\varvec{\nabla }_{\rho }{\hat{J}}$, the continuous dual operators $\left( \frac{\partial F}{\partial u}\right) ^{*}$ and $\left( \frac{\partial F}{\partial \rho }\right) ^{*}$ are needed. Starting with $\left( \frac{\partial F}{\partial u}\right) ^{*}$, first the operator $\frac{\partial F}{\partial u}$ has to be calculated using the definition of the Fréchet derivative

23 $$\begin{aligned} \frac{\partial F}{\partial u}h = \lim _{\epsilon \rightarrow 0^+} \frac{F\left( u\left( \rho \right) + \epsilon h, \rho \right) - F\left( u\left( \rho \right) , \rho \right) }{\epsilon } = \varvec{\nabla }\cdot \left[ \left( 1+\rho ^p\chi \right) \varvec{\nabla }h\right] \end{aligned}$$

Using the definition of dual operators in Hilbert space (18) to calculate $\left( \frac{\partial F}{\partial u}\right) ^{*}$ gives

24 $$\begin{aligned} \begin{gathered} \left( \frac{\partial F}{\partial u} w, z\right) = \int _{{\mathbb {R}}^3} \varvec{\nabla }\cdot \left[ \left( 1+\rho ^p\chi \right) \varvec{\nabla }w \right] \,z \,\text {d}V\\ = -\int _{{\mathbb {R}}^3} \left[ \left( 1+\rho ^p\chi \right) \varvec{\nabla }w \right] \varvec{\nabla }z \,\text {d}V=\int _{{\mathbb {R}}^3} w\,\varvec{\nabla }\cdot \left[ \left( 1+\rho ^p\chi \right) \varvec{\nabla }z \right] \,\text {d}V = \left( w, \left( \frac{\partial F}{\partial u}\right) ^*z\right) \text {,} \end{gathered} \end{aligned}$$

where partial integration is applied twice and the boundary integrals vanish if the same boundary condition as valid for the scalar potential *u* is also also demanded from *w* and *z*^18^.

The adjoint equation becomes

25 $$\begin{aligned} \varvec{\nabla }\cdot \left[ \left( 1+\rho ^p\chi \right) \varvec{\nabla }\lambda \right] = {\mathcal {R}}\left( \frac{\partial J}{\partial u}\right) \end{aligned}$$

with boundary condition

26 $$\begin{aligned} \lambda \left( {\varvec{r}}\right) \rightarrow {\mathcal {O}}\left( \frac{1}{\left| {\varvec{r}}\right| }\right) \quad \text {for} \quad \left| {\varvec{r}}\right| \rightarrow \infty . \end{aligned}$$

Note that this equation has the same form as the forward problem *F* itself. This is a consequence of the self-adjointness of the operator $\frac{\partial F}{\partial u}$ that is given since the above calculation shows that $\frac{\partial F}{\partial u} = \left( \frac{\partial F}{\partial u}\right) ^*$. Therefore, $\lambda$ also has to be twice differentiable and the adjoint equation can be solved by the same method as described in “Forward problem” section, as will be seen below in “Solving the adjoint equation” section.

The operator $\left( \frac{\partial F}{\partial \rho }\right) ^{*}$ is obtained by again first deriving the operator $\frac{\partial F}{\partial \rho }$ using the Fréchet derivative

27 $$\begin{aligned} \begin{aligned} \frac{\partial F}{\partial \rho }r&= \lim _{\epsilon \rightarrow 0^+} \frac{F\left( u\left( \rho \right) , \rho + \epsilon r\right) - F\left( u\left( \rho \right) , \rho \right) }{\epsilon }\\&=\varvec{\nabla }\cdot \left( p\rho ^{p-1}r\left[ \chi \left( \varvec{\nabla }u-{\varvec{H}}_{\text {ext}}\right) - {\varvec{M}}_{\text {r}}\right] \right) \text {.} \end{aligned} \end{aligned}$$

Using Eq. (18) gives

28 $$\begin{aligned} \begin{aligned} \left( \frac{\partial F}{\partial \rho }r, s\right)&= \int _{{\mathbb {R}}^3} \varvec{\nabla }\cdot \left( p\rho ^{p-1}r\left[ \chi \left( \varvec{\nabla }u-{\varvec{H}}_{\text {ext}}\right) - {\varvec{M}}_{\text {r}}\right] \right) s\,\text {d}V \\&=- \int _{{\mathbb {R}}^3} \left( p\rho ^{p-1}r\left[ \chi \left( \varvec{\nabla }u-{\varvec{H}}_{\text {ext}}\right) - {\varvec{M}}_{\text {r}}\right] \right) \cdot \varvec{\nabla }s\,\text {d}V \\&=- \int _{{\mathbb {R}}^3} r\left( p\rho ^{p-1}\left[ \chi \left( \varvec{\nabla }u-{\varvec{H}}_{\text {ext}}\right) - {\varvec{M}}_{\text {r}}\right] \right) \cdot \varvec{\nabla }s\,\text {d}V = \left( r, \left( \frac{\partial F}{\partial \rho }\right) ^{*}s\right) \text {.} \end{aligned} \end{aligned}$$

Here again partial integration was applied and the boundary integral vanished for *s* satisfying the boundary condition (26), which is fulfilled since in the expression for the gradient $\left( \frac{\partial F}{\partial \rho }\right) ^{*}$ is applied to $\lambda$ that also satisfies this boundary condition.

Finally, the gradient can be written as

29 $$\begin{aligned} \varvec{\nabla }_{\rho }{\hat{J}} = \left( p\rho ^{p-1}\left[ \chi \left( \varvec{\nabla }u-{\varvec{H}}_{\text {ext}}\right) - {\varvec{M}}_{\text {r}}\right] \right) \cdot \varvec{\nabla }\lambda + {\mathcal {R}}\left( \frac{\partial J}{\partial \rho }\right) \in R\text {.} \end{aligned}$$

### Solving the adjoint equation

In order to solve the adjoint Eq. (25) we first have a closer look at it. On the right hand side is the partial derivative of the objective functional *J* with respect to the scalar potential *u*. However, we will not directly impose conditions on the potential *u* but on the magnetic field ${\varvec{H}}$ that is defined as ${\varvec{H}}= - \varvec{\nabla }u$. Consider the weak form of the adjoint equation

30 $$\begin{aligned} \int _{{\mathbb {R}}^3} \varvec{\nabla }\cdot \left[ \left( 1+\rho ^p\chi \right) \varvec{\nabla }\lambda \right] \,v\,\text {d}V = \int _{{\mathbb {R}}^3} {\mathcal {R}}\left( \frac{\partial J}{\partial u}\right) \,v\,\text {d}V \end{aligned}$$

where *v* is a suitable test function. Performing partial integration on the left hand side and inserting $\frac{\partial J}{\partial u}v = \frac{\partial J}{\partial {\varvec{H}}}\frac{\partial {\varvec{H}}}{\partial u}v = -\frac{\partial J}{\partial {\varvec{H}}}\varvec{\nabla }v$ on the right hand side we get

31 $$\begin{aligned} -\int _{{\mathbb {R}}^3}\left( 1+\rho ^p\chi \right) \varvec{\nabla }\lambda \cdot \varvec{\nabla }v\,\text {d}V = -\int _{{\mathbb {R}}^3}{\mathcal {R}}\left( \frac{\partial J}{\partial {\varvec{H}}}\right) \varvec{\nabla }v\,\text {d}V\text {,} \end{aligned}$$

where the boundary term vanishes due to the boundary condition on $\lambda$. Assuming that the objective functional *J* is defined locally on an area $\Omega _{\text {opt}}$, this is the weak form of

32 $$\begin{aligned} \varvec{\nabla }\cdot \left( \left( 1+\rho ^p\chi \right) \varvec{\nabla }\lambda -{\mathcal {R}}\left( \frac{\partial J}{\partial {\varvec{H}}}\right) \right) = 0\text {,} \end{aligned}$$

that is identical to the forward problem in Eq. (7) with $\lambda$ instead of *u*, ${\mathcal {R}}\left( \frac{\partial J}{\partial {\varvec{H}}}\right)$ replacing $\rho ^p{\varvec{M}}_{\text {r}}$ and ${\varvec{H}}_{\text {ext}}= 0$. We can therefore use the same algorithm to solve the adjoint equation as is used to solve the forward problem. Note, that it is perfectly fine to assume the same jump conditions on $\lambda$ as on *u* since they are contained in the weak form of the forward problem anyways as shown in^19^.

### Obtaining the Riesz representers

To obtain the Riesz representers ${\mathcal {R}}\left( \frac{\partial J}{\partial {\varvec{H}}}\right)$ and ${\mathcal {R}}\left( \frac{\partial J}{\partial \rho }\right)$ appearing inside the adjoint equation and the expression for the gradient, the coefficient vectors ${\varvec{J}}_{{\varvec{H}}}$ and ${\varvec{J}}_{\rho }$ belonging to the functionals $\frac{\partial J}{\partial {\varvec{H}}}$ and $\frac{\partial J}{\partial \rho }$ respecively, are calculated using FEniCS^11^. These coefficient vectors can be used to calculate the action of the functionals onto an element of the primal space *r*^14^. For the functional $\frac{\partial J}{\partial \rho }$ this reads

33 $$\begin{aligned} \frac{\partial J}{\partial \rho }\left( r\right) = \left( {\varvec{J}}_{\rho },{\varvec{r}}\right) _{\ell ^2} = {\varvec{J}}_{\rho }^T{\varvec{r}}\text {,} \end{aligned}$$

where $\left( \cdot ,\cdot \right) _{\ell ^2}$ represents the euclidiean inner product. On the other hand, using the Riesz representer ${\mathcal {R}}\left( \frac{\partial J}{\partial \rho }\right)$ the action can be written as

34 $$\begin{aligned} \begin{aligned} \frac{\partial J}{\partial \rho }\left( r\right)&= \left( {\mathcal {R}}\left( \frac{\partial J}{\partial \rho }\right) , r\right) _{L^2} = \int _{\Omega }\sum _i {\mathcal {R}}_i\left( \frac{\partial J}{\partial \rho }\right) \psi _i\left( {\varvec{x}}\right) \sum _j r_j \psi _j\left( {\varvec{x}}\right) \;\text {d}{\varvec{x}}\\&=\sum _i {\mathcal {R}}_i\left( \frac{\partial J}{\partial \rho }\right) \sum _j r_j\int _{\Omega }\psi _i\left( {\varvec{x}}\right) \psi _j\left( {\varvec{x}}\right) \;\text {d}{\varvec{x}}= \varvec{{\mathcal {R}}}_{L^2}^T\left( \frac{\partial J}{\partial \rho }\right) M{\varvec{r}} \end{aligned} \end{aligned}$$

with $\psi _i\left( {\varvec{x}}\right)$ being the discrete basis functions and $M = \int _{\Omega }\psi _i\psi _j\,\text {d}V$ being the mass matrix. Since the two equations must deliver the same result and furthermore $M^T = M$, the Riesz representer with respect to the $L^2$ inner product can be obtained from the coefficient vector as

35 $$\begin{aligned} \varvec{{\mathcal {R}}}_{L^2}\left( \frac{\partial J}{\partial \rho }\right) = M^{-1}{\varvec{J}}_{\rho }\text {.} \end{aligned}$$

The Riesz representer ${\mathcal {R}}\left( \frac{\partial J}{\partial {\varvec{H}}}\right)$ is obtained in the same way.
